# Annotations

**Published:** 1888-10-06

**Authors:** 


### Annotations.

**Sir Morell Mackenzie's Book.**

The public and the medical profession are promised a work of a kind which we may safely say has never before been attempted. It is not an appeal from "Philip Drunk" to "Philip Sober," but nothing less than the taking of a case of a purely professional character from a professional tribunal to the larger tribunal of the civilised world. The step is a complete revolutionising of all medical ideas of propriety and etiquette. If any such course had been attempted in the case of an unknown and insignificant patient, the medical profession, by its various representatives, colleges of physicians and surgeons, newspapers, *Lancets*, and what not, would have been down upon the offending practitioner like a thousand outraged wolves ready to tear him to pieces. We shall be curious to see what the College of Physicians will have to say to one of its most distinguished fellows on the forthcoming unique and flagrant specimen of popular medical literature. If the College is wise it will have nothing at all to say. Medical etiquettes and codes of rules are all very well and very necessary for general guidance, and so long as they do not contravene common sense and public convenience it is well they should exercise a general controlling influence. But it must be understood that they are mere regulations of expediency, not moral laws, and that sometimes it is necessary to ignore them. The *Lancet* poses as a distinguished authority on all the little niceties of professional behaviour. What will it have to say to Sir Morell Mackenzie? It, of course, merely expresses the opinion of one or two anonymous persons who have earned no particular claim to consideration. The College of Physicians, on the other hand, represents the authority and dignity of the whole profession. Its behaviour concerns every medical man in the kingdom. We may hope and expect that, under its present President, who is an experienced man of the world as well as an eminent physician, it will take the large and statesmanlike view which the case demands. The position may be stated in a single sentence. All the world looked on with eager and sympathetic interest during the dead emperor's late illness; all the world was made acquainted with the progress of the case from day to day, and with the variety and conflict of opinion which it occasioned; all the world has heard of the charges of the medical profession in Germany; therefore it is right and proper that all the world should bear what can be said on the other side. Bodies of professional men are seldom distinguished for common sense and knowledge of the world. Everybody knows what Clarendon, the Tory historian, once said of the clergy. "The clergy," said he, "who know the least and take the worst measure of human affairs of all mankind who can write and read." We sincerely hope that, for the sake of the intelligence and honour of the medical profession, the College of Physicians will hold no meetings, and the *Lancet* will not wag its foolish tongue when the new book appears. It would be grievous to find that Clarendon's description of the clergy of a former age might be applied with full propriety to the medical profession of the present.

**Other People's Money.**

It is one of the easiest things in the world to spend money, especially other people's. That is surely one of the principal reasons why anything like a reasonable economy is seldom found at many hospitals and charitable institutions. There are only two sources of authority in any given hospital—the committee and its higher officials, especially the secretary and the matron. If a hospital is blessed with an economical secretary and a managing and saving matron, the institution will be prosperous and solvent, whatever be the character of the committee. But no hospital can rely upon having a secretary and matron, either or both of whom shall be economical; and hence the necessity for the committee to be always in a condition of intelligent watchfulness. What is most to be regretted in hospital officials is their want of sense. It seems to be a matter of no concern to many of them how money is spent, and whether the hospital is economically managed or not. They are always sure of their salaries, and their food and lodging. If funds are low, they do not suffer. It is the patients in the surrounding neighbourhood who have to bear the consequences of diminished resources. The great difficulty—nay, the impossibility of securing economy among hospital officials—lies in this, that no evil consequences fall upon themselves as the result of their extravagance. There is no personal loss, as there would be if they were spending their own money. Many of them have no regard for the honour of their institutions, no sense of shame. They have spent so long a period in constant begging that they have become by second nature beggars of the very sturdiest class. It is time that hospital committees recognised these facts. They should endeavour to meet these marked tendencies of official nature by attaching some severe penalty to proved extravagance, and some correspondingly considerable reward to proved economy combined with efficiency. Salaries should not be fixed, but movable; appointments should not be permanent, but only for a period, and pending thorough efficiency. Secretaries and matrons too often learn to look upon hospitals as their own peculiar preserves, and to regard committees as nuisances. All this must be changed, and changed soon. If the reckless extravagance which has been recently disclosed is seen to continue the public will do well to pick out some of the offending institutions, and to mark its sense of their stupidity and shamelessness by withholding its support entirely. When one or two hospitals shall have been made a conspicuous example of, others will take fright, and mend their ways.

**Examiners Examined at Last.**

The examination craze has held such complete sway over the official mind for so long a period that many had begun to despair of ever again seeing the dawn of a day of reason. But it is a long lane that has no turning, and, as is frequently the case, light has appeared in a most unexpected quarter. The other day, at Sheffield, Sir Andrew Clark is reported to have said to an assembly of medical students and teachers that examinations had now become so difficult, and questions so recondite, that there was a danger of its becoming impossible for any earnest, practical work to be done by medical students. Sir Andrew, if he is correctly reported, is of opinion that all the time of students is in danger of being spent in hunting up all sorts of out-of-the-way knowledge in all sorts of out-of-the-way books in order to make sure of passing their examinations by answering all sorts of out-of-the-way questions. Now is this so? If it be, medical examiners must be even greater fools than ordinary professional persons. We do not speak in the interests of medical students, but of medical education and of those on whom medical students will ultimately be licensed to practise their art. What, in the name of common sense, is the object of medical education? Is it not to teach men to understand and to manage human creatures in a diseased condition? There are certain broad and unvarying facts underlying this art; certain principles also which are definite and unchanging. These are the foundations on which every medical man's education must be built; these the indispensables which every student must be well and thoroughly taught; these the regions through which every candidate must necessarily be taken by his examiners. The object of medical education should be to make a man thoroughly acquainted with all the familiar and necessary things; and if these things, by long familiarity, become the commonplaces of the class-room and of the smoking-room so much the better, because they are the very essence of that knowledge which is required in every-day practice. Of course, for the purpose of ascertaining the comparative diligence and ability of men, wider ranges may be included and profounder deeps explored both in lecture-room and examination-hall. But every student should have the assurance that an intelligent understanding of the great common facts and principles of medical knowledge and practice will at least secure him a "pass" at the critical moment of his life. If this were the case we should gain two distinct and worthy results; there would be fewer instances of broken-down and mentally-enfeebled students; and there would be a greatly-heightened practical efficiency among ordinary practitioners. Among the innumerable ills with which human society is cursed there are few which work more harm than prigs and pedants.

